# Impact of Neoadjuvant Induction Chemotherapy Prior to Chemoradiation on Survival and Surgical Outcomes in Real-World Esophageal Adenocarcinoma Cohort

**DOI:** 10.3390/cancers18020213

**Published:** 2026-01-09

**Authors:** Thomas M. Matoska, Abdullah A. Memon, Lou-Anne Acevedo Moreno, Calista Bulacan, Lisa Rein, Anjishnu Banerjee, Ben George, Lauren Jurkowski, Alexandria Phan, Candice Johnstone, Monica E. Shukla, Elizabeth M. Gore, Paul Linsky, Mario Gasparri, Mallory Hunt, Lindsay L. Puckett

**Affiliations:** 1 Department of Radiation Oncology, Medical College of Wisconsin, 8701 Watertown Plank Road, Milwaukee, WI 53226, USA; tmatoska@mcw.edu (T.M.M.);; 2 Department of Biostatistics, Medical College of Wisconsin, 8701 Watertown Plank Road, Milwaukee, WI 53226, USA; 3 Department of Medical Oncology, Mayo Clinic Comprehensive Cancer Center, 200 First St. SW, Rochester, MN 55905, USA; 4 Department of Internal Medicine, Endeavor Health, 2650 Ridge Ave., Evanston, IL 60201, USA; 5 Department of Medicine, Division of Hematology and Oncology, Medical College of Wisconsin, 8701 Watertown Plank Road, Milwaukee, WI 53226, USA; 6 Department of Surgery, Division of Cardiothoracic Surgery, Medical College of Wisconsin, 8701 Watertown Plank Road, Milwaukee, WI 53226, USA

**Keywords:** esophageal cancer, esophageal adenocarcinoma, induction chemotherapy, chemoradiation

## Abstract

Background/objectives: Improvements in esophageal adenocarcinoma (EAC) treatment have reduced mortality. While chemoradiation before surgery was previously a standard of care, updated guidelines recommend peri-operative chemotherapy without chemoradiation. Continued investigation into optimal non-operative treatment paradigms for patients who defer surgery or are not candidates for surgery and certain chemotherapy regimens is needed. The impact of induction chemotherapy prior to chemoradiation on survival and surgical outcomes remains unclear. This study assessed survival and surgical outcomes in a real-world cohort of EAC patients receiving induction chemotherapy before chemoradiation. Methods: This single-institution, IRB-approved, retrospective cohort study included patients with newly diagnosed stage II-IVb (oligometastatic for IVb) EAC who received definitive chemoradiation (radiation ≥ 40 Gy and two cycles of chemotherapy) +/− esophagectomy from 2007 to 2022. Patients receiving induction chemotherapy were compared to those who did not. Endpoints included survival and surgical outcomes. Results: A total of 141 EAC patients received definitive chemoradiation; 83 received induction chemotherapy before chemoradiation. Patients receiving induction chemotherapy were younger (*p* < 0.01) with slightly lower performance status (*p* = 0.27) and presented at a more advanced stage (*p* < 0.001). Median OS was 3.5 years in the induction chemotherapy group compared to 2.2 years (*p* = 0.10). There was no difference in pathologic complete response (*p* = 0.81), esophagectomy frequency (*p* = 0.87), or surgical downstaging between treatment groups (*p* = 0.84). Conclusions: In this real-world, single-institutional patient cohort investigating induction chemotherapy prior to chemoradiation in EAC, patients receiving induction chemotherapy did well but did not have a statistically significant improvement in survival outcomes or surgical outcomes. This study showed that significant numbers of real-world patients may not receive esophagectomy. Thus, prospective, randomized clinical trials are warranted to better delineate the efficacy and selection of patients for induction chemotherapy when non-operative approaches are favored.

## Simple Summary

The current standard of care for esophageal cancer is typically peri-operative chemotherapy and surgery. However, there are patients who prefer not to receive surgery or are not candidates for surgery or certain chemotherapies. Thus, non-operative approaches to esophageal cancer should continue to be studied. Chemoradiation can be part of non-operative approaches, and the role for chemotherapy before chemoradiation is not well-defined. The purpose of this study is to report outcomes of those who received induction chemotherapy (as compared to those who did not) prior to chemoradiation with or without surgery in a real-world population.

## 1. Introduction

Esophageal cancer accounts for about 16,250 deaths each year in the United States. Encouragingly, survival outcomes have been slowly improving due to better treatment regimens [1,2]. The CROSS trial set a previous standard of care option for locally advanced esophageal adenocarcinoma (EAC) with preoperative chemoradiation (CRT) followed by surgery [3]. While the CROSS regimen was a main standard of care option, 5-year survival rate for esophageal cancer was around 20% for all stages [4]. More recently, the ESOPEC trial showed a survival benefit in patients receiving peri-operative FLOT (5-FU, leucovorin, oxaliplatin, docetaxel) chemotherapy (CHT) and surgery compared to CRT and surgery. Given these results, NCCN guidelines now recommend peri-operative CHT as the preferred treatment paradigm for locally advanced EAC [5,6]. However, the guidelines detail that upfront CRT can be indicated for patients who decline or are medically unfit for surgery, borderline resectable tumors, and poor candidates for FLOT CHT. In clinical practice, this represents a meaningful proportion of patients.

The role of induction chemotherapy prior to CRT in EAC is not well-defined. Currently, NCCN guidelines state induction CHT prior to CRT in EAC can be considered in certain cases such as relieving dysphagia, but phase III data is required for further recommendations [6]. Potential advantages of induction CHT prior to CRT include tumor downstaging, improved resectability, and reduced rates of local and distant failure; however, these advantages have not been consistently proven in the literature and data is lacking regarding ideal induction CHT regimens [7]. For EAC, induction cisplatin/5-FU prior to CRT had higher overall survival (OS) although this was not statistically significant compared to peri-operative chemotherapy [8]. Induction FOLFOX (leucovorin, fluorouracil [5-FU], and oxaliplatin) has been shown to improve near pathologic complete response rates (pCR) compared to patients treated with CRT alone [9]. One phase II trial showed improvement in OS with induction CHT in well or moderately differentiated tumors [10]; however, another phase II trial only found improvements in pCR rates and not OS with induction CHT + CRT compared to neoadjuvant CHT alone [11]. Additionally, a national cancer database study found improved survival in patients who received induction CHT prior to CRT [12]. Conversely, one small single-institutional retrospective study (*n* = 95) investigating induction CHT found that patients who received induction CHT had numerically lower survival [13].

In the new age of peri-operative CHT being the standard of care for EAC, continued study into optimal non-operative approaches, such as CRT +/− induction CHT, is warranted because not every patient is a candidate for or is willing to recieve an esophagectomy in real-world settings. Within our study, we sought to investigate survival and surgical outcomes in patients with stage II-IVb (IVb: oligometastatic only) EAC treated with definitive intent CRT with or without induction CHT and with or without esophagectomy in real-world clinical practice. Prior to results analysis, we hypothesized induction CHT would lead to improved survival and surgical (surgical downstaging, pCR) outcomes.

## 2. Materials and Methods

This Institutional Review Board approved single-institution study was conducted from 2022 to 2024 via retrospective review of electronic medical records. We identified treatment naïve, newly diagnosed patients with Stage II to oligometastatic stage IVB (≤5 sites of metastasis, including non-regional lymph nodes) (AJCC 8th ed.) EAC at diagnosis [14]. Histologic diagnosis was determined by our institution’s pathology department via esophageal biopsy. Patients treated with definitive intent CRT (radiation dose ≥ 40 Gy with at least two cycles of induction or concurrent CHT) with or without esophagectomy between 2007 and 2022 were included. Individual treatment paradigms were determined by medical oncologists, radiation oncologists, and thoracic surgeons in a multidisciplinary tumor board setting. Factors such as performance status, extent of disease, comorbidities, symptoms at diagnosis such as dysphagia, and patient motivation were considered for induction CHT use. Surgical candidacy was determined by multidisciplinary tumor board and formal thoracic surgery evaluation that occurred either before any treatment or early on in neoadjuvant/definitive treatment. Considerations for surgery included factors such as age, performance status, comorbidities, and patient preference. The following exclusion criteria were applied: no induction chemotherapy or CRT given at our institution, palliative radiation doses, insufficient follow-up or patient information available in the electronic medical record, non-adenocarcinoma histology, and prior diagnosis of esophageal cancer with prior definitive treatment. Final sample size was determined by the number of patients who met inclusion criteria.

After the patient selection process was completed, patients were separated into two groups: those who received induction CHT (at least one cycle of CHT prior to CRT) and those who did not receive induction CHT. For all patients, collected variables from the electronic medical record included age at diagnosis, clinical and pathologic staging, ECOG performance status prior to the start of any treatment, number of induction CHT cycles, induction CHT regimen, CRT regimen and dose, receipt of adjuvant immunotherapy (nivolumab or pembrolizumab), date of diagnosis, date of death, and date of last follow-up. Investigated endpoints included survival outcomes such as OS and progression-free survival (PFS), as well as surgical outcomes. Surgical outcomes included esophagectomy frequency, pCR (no residual disease on surgical pathology), and surgical downstaging (decrease in T stage or N stage from diagnosis to pathologic staging at surgery). Descriptive safety measure data collected for each patient included radiation dose reductions from originally prescribed dose, overnight hospital admissions during or within 30 days after completing induction CHT and CRT, and overnight hospital admissions within 90 days of esophagectomy. Descriptive statistics were reported for all study variables. Frequency and percentage were used to summarize categorical variables. Continuous variables were summarized with mean, median, standard deviation, and range.

Univariate analyses (UVA) and a multivariate analysis (MVA) Cox proportional hazards regression model were performed to evaluate OS. Variables included for MVA to account for bias or confounding variables were age, stage, esophagectomy (time dependent), ECOG performance status, receipt of adjuvant immunotherapy (pembrolizumab or nivolumab, time dependent), and receipt of induction CHT. A Cox proportional hazard model was performed to evaluate induction chemotherapy effect on OS in subgroup of patients who did have esophagectomy and a subgroup in those who did not have esophagectomy.

The Kaplan–Meier method was used to describe OS and PFS [15]. The survival time was considered the time from diagnosis to the time of the event (death for OS, or relapse/progression for PFS). Unadjusted comparisons of OS and PFS between treatment groups were performed using the log-rank test; adjusted comparisons of OS and PFS for categorical variables and comparisons of survival curves between continuous variables were analyzed using the Cox proportional hazards regression [16]. No adjustments were made for multiple testing. Comparison of surgical downstaging and pCR rates between treatment groups was performed using Chi Squared tests. Unadjusted incidence of esophagectomy frequency between treatment groups was performed using Gray’s test [17]. Statistical analyses were performed utilizing R version 4.4.1 [18]. If data was missing for a certain variable for patients, this patient was excluded from that specific analysis, but not the entire cohort. The manuscript was written in accordance with the Strobe declaration.

## 3. Results

### 3.1. Patient Characteristics

**Figure 1:**
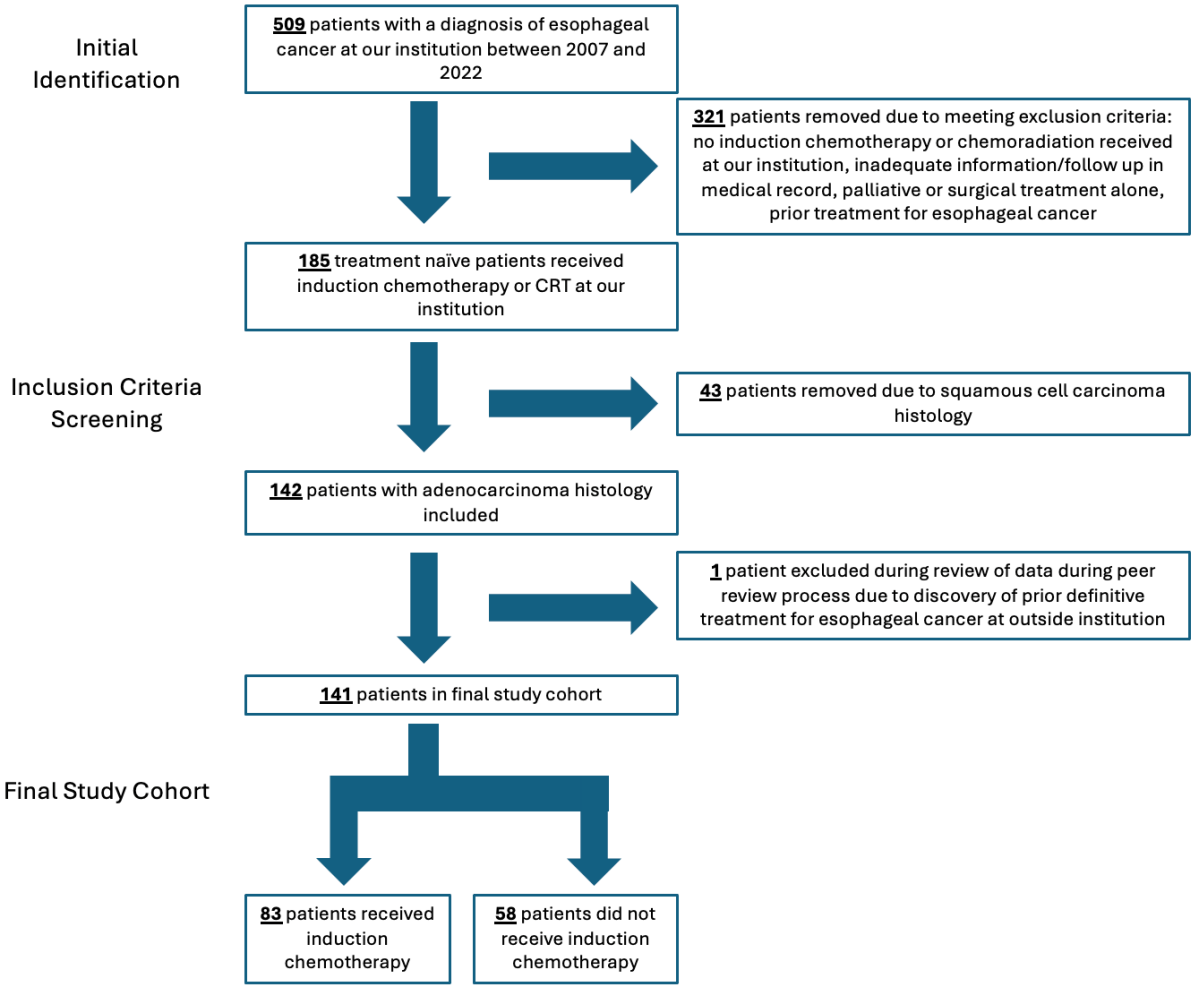
Flowchart that provides overview of this study’s inclusion process.

A total of 185 patients at our institution received definitive CRT during the study period and had sufficient information for inclusion; 141 EAC patients were included in the analysis. Of these, 83 (59%) received induction CHT prior to CRT (Figure 1). Median follow-up for all patients (time to last follow-up or death) was 3.1 years, and median follow-up for patients still alive was 7.5 years. The mean age at diagnosis was 64.3 years for patients receiving induction CHT and 70.0 years in patients treated without induction CHT (*p* < 0.01), and patients were predominantly male in both groups (*n* = 129, 91%). Mean ECOG performance score was slightly lower in the induction CHT group (0.56 vs. 0.72, *p* = 0.272). A greater proportion of patients in the induction CHT group had a more advanced stage disease at diagnosis (36.1% stage IV disease in the induction CHT group vs. 10.3%) (*p* < 0.001). In the induction CHT group, 24 patients (28.9%) received pembrolizumab or nivolumab for any reason compared to 8 patients (13.8%) in those who did not receive induction CHT. Baseline characteristics are summarized in Table 1.

### 3.2. Induction Chemotherapy Characteristics

The most common induction CHT regimens were FOLFOX (*n* = 31) and modified DFOX/FLOT (*n* = 33). Of patients in the induction cohort, 13 patients received trastuzumab as part of their induction therapy. The mean number of cycles of induction CHT was 4.45 cycles (SD 3.04, range 1–23 cycles). There were no significant differences in OS and PFS according to the type of induction CHT regimen (OS: *p* = 0.45, PFS: *p* = 0.86) or the number of CHT induction cycles (OS: *p* = 0.96, PFS: *p* = 0.58). Induction CHT characteristics are summarized in Table 2.

### 3.3. Surgical Outcomes

The frequency of esophagectomy was 56.6% (*n* = 47) in patients who received induction CHT compared to 48.3% (*n* = 28) in patients treated without induction CHT (*p* = 0.87). One patient who did not receive induction CHT did not have surgical pathology information available and was excluded from pCR and downstaging calculations. pCR was seen in 23% (*n* = 11) with induction CHT versus 26% (*n* = 7) (*p* = 0.81). Surgical downstaging (decrease in T or N stage) was 68% for induction CHT (*n* = 32) and 70% (*n* = 19) for those treated without induction CHT (*p* = 0.84).

### 3.4. Survival Outcomes

**Figure 2:**
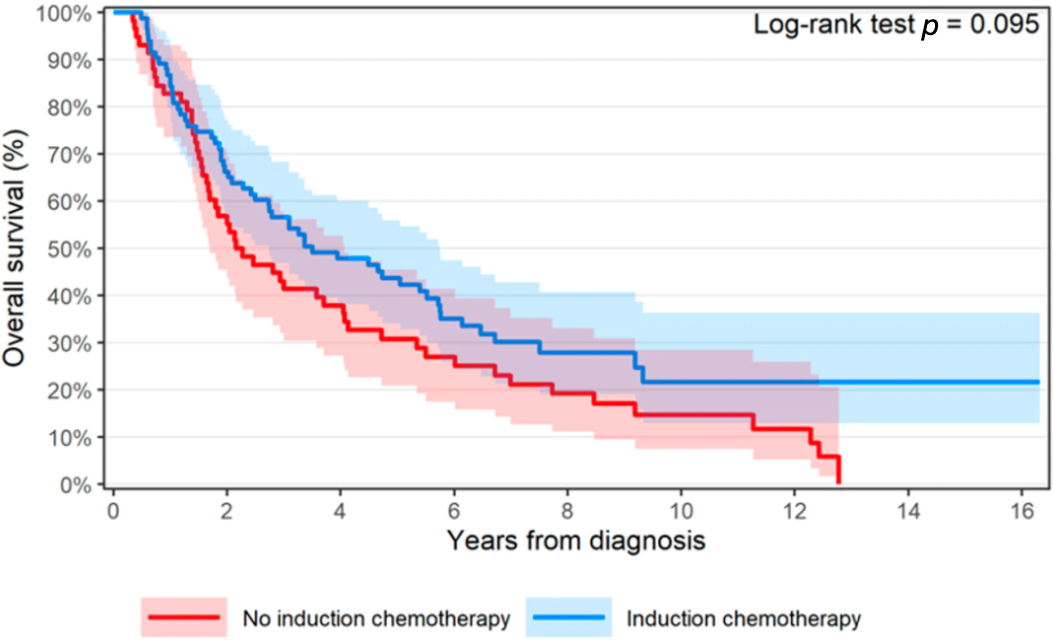
Survival graph comparing patients with induction CHT (blue) and without (red).

**Figure 3:**
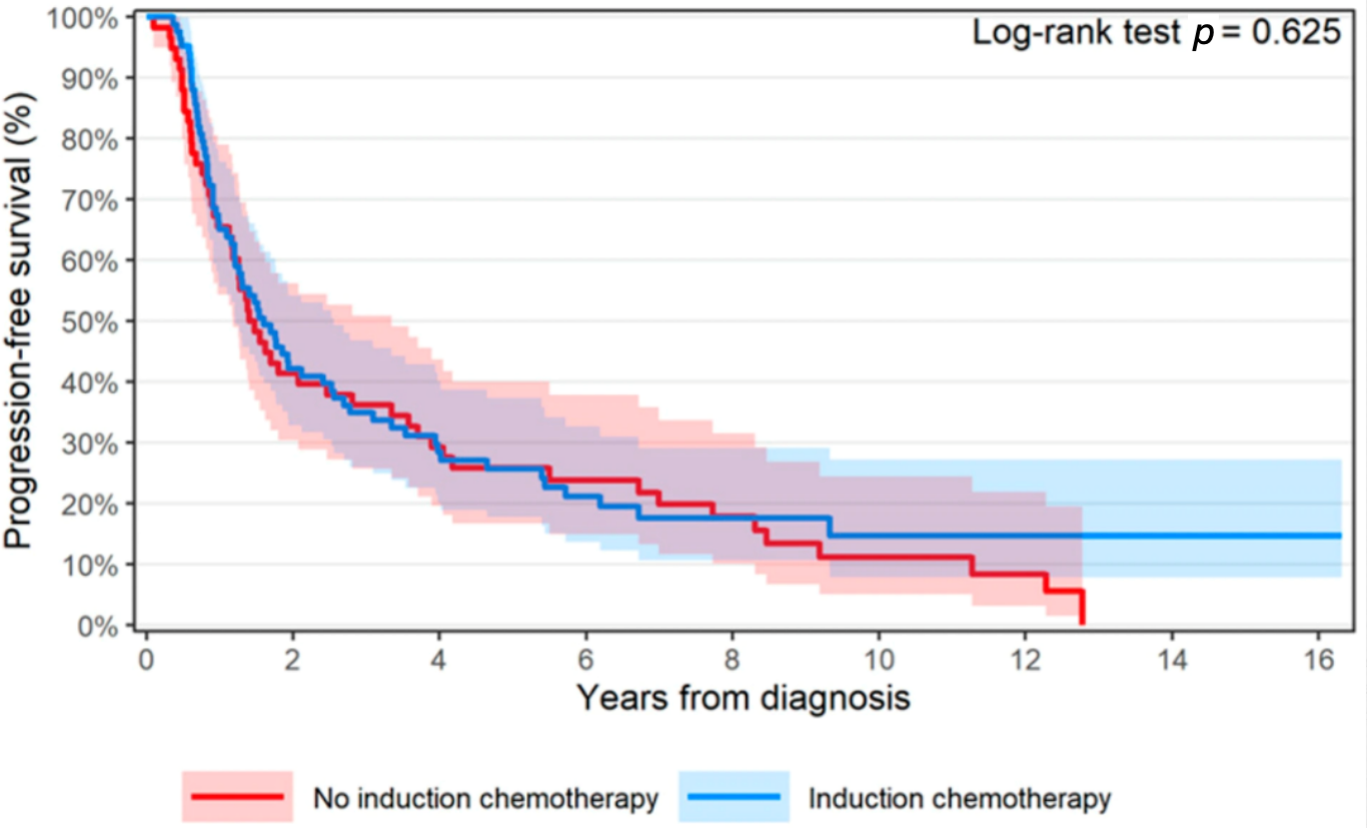
Survival graph shows progression-free survival for patients with induction CHT (blue) versus those without (red).

All patients had survival data available. The 3- and 5-year OS were 56.6% [46.9–68.4%, 95% CI] and 43.7% [34.1–56.0%, 95% CI] with induction CHT, compared to 43.1% [32.1–57.9%, 95% CI] and 30.8% [20.9–45.4%, 95% CI] without. OS favored induction CHT with a median OS of 3.50 [2.73–5.75 95% CI] vs. 2.21 years [1.69–4.07 95% CI], but there was no statistically significant difference (*p* = 0.10, univariate analysis) (Figure 2). Multivariate analysis showed lower ECOG PS was significantly associated with better OS, but induction CHT (*p* = 0.052, HR 0.64 [0.41–1.00 95% CI]) was not (Table 3). Immunotherapy use for any reason (nivolumab/pembrolizumab) was associated with worse survival on multivariate analysis (*p* < 0.001). For all included patients, median PFS was 1.60 [1.21–2.56 95% CI] in the induction CHT group vs. 1.43 years [1.18–3.34 95% CI] (*p* = 0.63) (Figure 3). In a subgroup analysis of patients who received esophagectomy, there was no difference in OS when comparing induction CHT use (*p* = 0.57, HR 0.85 [0.5–1.46 95% CI]). No significant difference was seen in induction CHT use vs. no induction CHT use in those who did not receive esophagectomy (*p* = 0.073, HR 0.61 [0.36–1.05 95% CI]).

### 3.5. Descriptive Safety Measures

All patients in our cohort were able to complete a definitive dose of radiation (≥40 Gy). There were 5 patients (6.0%) in the induction chemotherapy group who had their originally prescribed radiation dose reduced during chemoradiation compared to 2 patients (3.4%) in the group who did not receive induction chemotherapy. Of patients receiving induction chemotherapy, 7 patients (7.4%) were hospitalized during or 30 days after induction chemotherapy. When looking at admissions within 90 days of esophagectomy, 14 patients (29.8%) in the induction chemotherapy group had at least one overnight hospital admission compared to 7 (25%) in those who did not receive induction CHT. For admissions during or within 30 days of completion of CRT, 14 patients (16.9%) in the induction chemotherapy group and 21 patients (36%) in the no induction chemotherapy group had at least one overnight hospital admission.

## 4. Discussion

For patients with resectable locally advanced EAC, NCCN guidelines give preference to peri-operative CHT over neoadjuvant chemoradiotherapy based on the results of the ESOPEC trial. The role for induction CHT prior to neoadjuvant and/or definitive CRT is not well-defined, and investigation into optimal non-operative treatment is warranted for patients who are not candidates for peri-operative CHT regimens (FLOT), poor surgical candidates, or patients who defer surgical approaches. In this single-institutional, real-world cohort of EAC patients receiving definitive CRT with or without surgery, patients who received induction CHT did not have statistically different survival outcomes (OS and PFS) and surgical outcomes (pCR, completed esophagectomy, surgical downstaging [decrease in T or N stage from clinical staging to pathologic staging]). When looking at OS, 3- and 5-year OS in patients receiving induction CHT were 56.6% and 43.7% compared to 43.1% and 30.8% in patients who did not receive induction CHT (*p* = 0.10, univariate analysis). Notably, patients receiving induction CHT were younger (*p* < 0.01) and had slightly lower average PS (0.56 vs. 0.72, *p* = 0.27) compared to patients not receiving induction CHT. However, induction CHT was utilized in patients who presented at more advanced stages (stage III, IVA, IVB) (*p* < 0.001), which could impact the OS, PFS, and surgical outcomes. More patients in the induction CHT cohort received adjuvant pembrolizumab/nivolumab (28.9% vs. 13.8%); however, this was associated with survival detriment on MVA, likely due to immunotherapy often being used in setting of recurrence. Despite the complex differences between favorable and unfavorable characteristics in this patient cohort, these results raise the possibility that induction CHT may improve OS in patients with locally advanced EAC if studied in a more controlled setting such as a dedicated trial.

Table 4 reviews treatment regimens, inclusion criteria, and OS outcomes from landmark EAC trials and our real-world retrospective cohort [3,5,19,20]. While these trials are provided for reference, comparison between trials is limited and cautioned, given differences in study populations, inclusion criteria, and treatment techniques. The CROSS trial, which did not utilize induction CHT, and established the previous standard of care of CRT followed by surgery, varied substantially from our population in that it included squamous cell carcinoma (SCC) histology and included patients with less advanced disease compared to this study’s cohort. These are important to consider when comparing 5-year OS from CROSS (47%) to our study’s group of patients receiving induction CHT (44%) [3]. In real-world settings, the CROSS regimen has been adopted to include more advanced nodal involvement and even oligometastatic patients [21], and our study reflects this broader application of CRT with or without surgery.

In our study, induction chemotherapy did not show a statistically significant benefit for OS (*p* = 0.052 on MVA, *p* = 0.10 on univariate) and surgical outcomes, which could be due to reasons such as an underpowered study, heterogeneous patient cohort with confounding factors that are not equal between treatment groups, or a true lack of benefit of induction CHT. There may also be subgroups of patients that would benefit from induction CHT, many of which are not included in our study. Our study did suggest the benefit of induction at our institution may be greater in those who are not surgical candidates in sub group analysis based on surgical status (*p* = 0.073, HR 0.61 [0.36–1.05 95% CI]). In the current literature, induction CHT use for EAC remains heterogeneous, lacks consensus, and is largely limited to phase II and retrospective data. The phase II trial CALGB 80803 evaluated induction CHT prior to CRT, using PET response to guide subsequent concurrent CHT choice. For all patients receiving induction CHT, the 5-year OS was 44.9%, similar to this study’s induction CHT group [19]. One phase II trial comparing induction docetaxel, oxaliplatin, and capecitabine prior to CRT and surgery to no induction CHT and showed longer median OS in well and moderately differentiated tumors [10]. A United States National Cancer Database study (*n* = 12,460, 11,880 received CRT and 580 received induction CHT + CRT) found a statistically significant improvement in OS in patients receiving induction CHT before CRT [12]. Conversely, a recent single-institutional retrospective study found no difference in OS when investigating induction CHT [13]. To our knowledge, there are no phase III studies investigating induction CHT compared to no induction CHT prior to CRT in EAC patients.

There is a lack of consensus on what kind of induction CHT regimen and the number of inductions CHT cycles used in EAC. This is evidenced by the heterogeneity of induction CHT used in our study. FOLFOX and FLOT were the two most common regimens used, and there was no difference in OS among different induction CHT regimens or the number of cycles of induction CHT between the two patient cohorts. One of the only trials to compare induction CHT regimens is the previously discussed CALGB 80803, which suggested patients who received FOLFOX induction CHT and FOLFOX concurrent CHT had better responses compared to carboplatin-paclitaxel [19]. Single-arm induction CHT trials have investigated other regimens, including 5-FU/oxaliplatin and docetaxel/oxaliplatin/capecitabine [10,22,23]. Studies in gastric cancer, gastro-esophageal junction, and EAC have investigated peri-operative CHT regimens without CRT. FLOT has been shown to benefit patients receiving peri-operative CHT and surgery in both gastric and, more recently, EAC [5,24].

This study’s patient cohort (2007–2022) predates the trials that reshaped the treatment paradigm shift for EAC. The ESOPEC trial showed an OS and PFS benefit for patients receiving peri-operative FLOT and surgery compared to CRT and surgery [5]. The Neo-AEGIS phase III trial also demonstrated non-inferiority for peri-operative CHT compared to neoadjuvant CRT [20]. In comparison with our induction CHT cohort, the ESOPEC peri-operative FLOT and surgery arm had comparable 3-year OS (57.4% vs. 56.6%) but higher 5-year OS (50.6% vs. 43.7%). Direct comparisons between our study and ESOPEC should not be made. Our study included a real-world patient cohort outside of a clinical trial setting, oligometastatic patients, many patients who did not undergo esophagectomy, and was subject to selection bias due to its retrospective nature. The MATTERHORN trial was built on predecessor trial paradigms and combined peri-operative FLOT with or without durvalumab. Patients who received durvalumab and peri-operative FLOT had significantly higher OS and event-free survival [25]. It is essential to view our results in the context of the evolving role of immunotherapy. Because our cohort includes patients diagnosed as early as 2007, many patients did not receive modern immunotherapy for indications like recurrence or as regular adjuvant therapy as per KEYNOTE 061, which could affect this study’s outcomes and comparisons to modern trials [26]. When assessing immunotherapy use in our cohort on OS multivariate analysis, there was a significant survival detriment, likely due to immunotherapy being used for recurrence. Nonetheless, this study represents a real-world, less-discussed cohort of patients who may not want surgery or may be poor surgical candidates at diagnosis, and these patients can still achieve reasonable OS outcomes with induction CHT and definitive CRT.

Related to surgical outcomes in our real-world cohort, there was no significant difference in pCR (*p* = 0.81), pathological downstaging (*p* = 0.84), or esophagectomy frequency (*p* = 0.96) between those who received induction CHT and those who did not. One possible explanation is that the induction CHT cohort had more advanced staging at diagnosis, thus making esophagectomy or pCR at surgery somewhat less likely from the start. Alternatively, it may be that induction CHT does not provide enough tumor downstaging and complete response benefit to be appreciated in this cohort size (75 patients [52%] had surgery among both cohorts). Yoon et al. evaluated induction CHT followed by CRT and found no improvement in pCR or resectability [10]. One national database study found improvements in pCR with induction CHT in a subgroup of patients treated in the latter half of the study time period (2013–2015) [12]. Another retrospective (*n* = 205) study showed no difference in pCR outcomes [27]. The phase III TOPGEAR trial investigated CRT and peri-operative CHT compared to peri-operative CHT alone and found improved rates of pCR in the CRT and peri-operative CHT arm, but no significant difference in OS [28]. Despite these studies, surgical outcomes may not comprehensively inform us on the value of induction CHT, and survival data remains an essential part of this discussion.

Descriptive safety measure data from our study should be interpreted with caution. There were similar rates of dose reductions in radiation among induction CHT and no induction CHT cohort (6% vs. 3.4%). Hospitalizations for each treatment group during CRT and induction CHT along with after surgery were reported, but in retrospective studies, cause of side effects due to specific treatment cannot be directly confirmed like in prospective studies. A small number of patients were hospitalized during induction CHT (7.4%), and there were similar amounts of hospitalizations within 90 days of surgery in the induction CHT group (29.8%) and group without induction CHT (25%). Patients who did not receive induction CHT had a notably higher number of admissions during CRT (36%) than those who did receive induction CHT (17%). This could be due to induction CHT relieving symptoms of induction chemotherapy such as dysphagia as recommended by NCCN guidelines. Alternatively, it is possible that at baseline the patients without induction CHT had comorbidities that predisposed them to hospitalization during CHT. Prospective studies would more reliably report safety events compared to our study.

This study only included EAC patients. SCC and EAC have significant genomic differences that could affect treatment response and tumor behavior [29]. Induction CHT data for SCC also remains heterogeneous. One phase III trial investigated induction CHT + CRT compared to CRT alone in SCC, finding no difference in OS or PFS. This trial, however, performed an exploratory gene expression analysis that found six genes that predicted for efficacy of induction CHT [30]. No phase III EAC studies of this nature exist to our knowledge, but we hope our study can encourage further prospective investigation into efficacy and selection of patients receiving induction CHT prior to CRT for EAC.

Our study has several limitations. In clinical practice, higher-risk or more advanced-stage patients were more likely to receive induction CHT based on physician judgment and, therefore, lead to some selection bias especially given our study’s retrospective nature. Also, the patients who received induction CHT had lower ECOG PS at diagnosis (*p* = 0.27) and were younger (*p* < 0.01), which could affect survival in favor of induction chemotherapy. Our study was conducted within a single institution, making our results difficult to apply to outside institutions and other populations of patients. Additionally, patients who received induction CHT but progressed prior to completing CRT were excluded from this study, which may skew our findings in favor of the induction CHT group. This exclusion introduces the potential for immortal time bias, as patients needed to survive long enough to receive both induction CHT and CRT to be included in the analysis. However, induction CHT can be considered a meaningful part of these patients’ survival. Lastly, our study may be underpowered due to limited sample size. We acknowledge this retrospective study is hypothesis generating and meant to inspire and support future prospective and multi-institutional studies. Thus, conclusions from the study should be interpreted with caution due to the aforementioned limitations.

Ongoing clinical trials are exploring neoadjuvant systemic treatment strategies in EAC and gastroesophageal carcinoma. One ongoing phase II study is evaluating the use of induction FLOT followed by neoadjuvant CRT in resectable EAC [31]. The CRITICS-2 study is a phase II trial that seeks to compare neoadjuvant DOC CHT (docetaxel + oxaliplatin + capecitabine) to neoadjuvant induction DOC and neoadjuvant CRT prior to surgery in gastric and gastroesophageal junction adenocarcinoma [32]. Multiple studies aim to reduce radiation toxicity, which could increase definitive CRT use in patients who are not optimal surgical candidates. A phase IIB study compared proton beam radiation therapy to intensity modulated photon radiation therapy, and results showed reduced toxicity with proton therapy [33]. Additionally, another study aims to decrease CRT toxicity with pulsed-low dose rate for esophageal cancer [34].

## 5. Conclusions

In conclusion, EAC patients receiving induction CHT prior to CRT had numerically higher OS (5-year OS 44% vs. 31%, *p* = 0.10 univariate analysis) and rates of esophagectomy (56.6% vs. 48.3%), but there was no statistically significant improvement in any survival or surgical outcome in our real-world, heterogeneous patient cohort. A significant amount of patients did not undergo standard of care esophagectomy in this study. Thus, further research on induction CHT and neoadjuvant/definitive CRT is needed, especially for those who defer or are unfit for surgery or certain CHT regimens. Prospective, randomized controlled phase III trials on induction CHT for EAC are essential to assess its benefits. Studies should also identify candidacy and responders to induction CHT, like validated biomarkers.

## Figures and Tables

**Table 1:** Table comparing demographic information between the study’s treatment cohort.

	Induction CHT (*n* = 83)	No Induction CHT (*n* = 58)	*p*-Value
Mean age at diagnosis (years) [Range]	64.3 [40–84]	70.0 [45–89]	*p* < 0.01
Male	77 (93%)	52 (90%)	*p* = 0.55
Average ECOG PS [Range]	0.56 (*n* = 3 missing) [0–2]	0.72 (*n* = 4 missing) [0–3]	*p* = 0.27
Median OS (years) [95% CI]	3.50 [2.73, 5.75]	2.21 [1.69, 4.07]	*p* = 0.10
Stage II	4 (4.8%)	10 (17.2%)	*p* < 0.001
Stage III	49 (59.0%)	42 (72.4%)	-
Stage IV	30 (36.1%)	6 (10.3%)	-
Esophagectomy	47 (56.6%)	28 (48.3%)	*p* = 0.87

Abbreviations: CHT = Chemotherapy; PS = Performance Status; OS = Overall Survival.

**Table 2:** Table outlining the type and number of cycles of induction chemotherapy in our patient cohort.

Subset of Patients Who Received Induction Chemotherapy	
Characteristic	*n* = 83 ^1^
Induction chemo type	
FLOT	33 (40%)
FOLFOX	31 (37%)
Other	19 (23%)
Herceptin use at induction	
No	69 (84%)
Yes	13 (16%)
Missing	1
Number of induction cycles	
Mean (SD)	4.45 (3.04)
Median [Q1, Q3]	4.00 [2.00, 5.00]
Min, Max	1.00, 23.00
Number of induction cycles	
1–3 cycles	33 (40%)
4–8 cycles	45 (54%)
9+ cycles	5 (6%)

^1^ *n* (%).

**Table 3:** Results of multivariate analysis for overall survival in patients receiving chemoradiation. Higher ECOG performance significantly links to worse survival.

Characteristic	HR	95% CI	*p*-Value
Induction chemotherapy			
No	—	—	
Yes	0.64	0.41, 1.00	0.052
Age (units of 10 years)	1.00	0.79, 1.25	0.966
Stage			
II	—	—	
III	1.43	0.72, 2.87	0.309
IVA	3.15	1.31, 7.57	0.010
IVB	1.11	0.42, 2.93	0.837
ECOG			
0	—	—	
1	1.64	1.07, 2.51	0.024
2–3	4.20	2.04, 8.65	<0.001
Unknown	1.73	0.67, 4.52	0.260
Surgery (time dependent)			
No	—	—	
Yes	0.90	0.53, 1.53	0.699
Immunotherapy (time dependent)			
No	—	—	
Yes	2.84	1.68, 4.79	<0.001

Abbreviations: CI = Confidence Interval, HR = Hazard Ratio.

**Table 4:** Compares OS outcomes from landmark EAC trials and our current retrospective study.

Study	Histology	Regimen	Median OS	3-Year OS	5-Year OS	Stage Included	ECOG Range
Matoska et al. (induction arm)	Adenocarcinoma	Induction CHT + CRT +/− esophagectomy	3.5 years	57%	44%	T1N1-3M0, T2-4NanyM0, TanyNanyM1 (oligometastatic)	0–2
CROSS	Adenocarcinoma and squamous cell carcinoma	CRT + esophagectomy	4.1 years	58%	47%	T1N1 or T2-3N0-1M0	0–2
CALGB 80803	Adenocarcinoma	Induction CHT + CRT + esophagectomy	4.1 years	N A	53%	T1N1-3M0, T2-4NanyM0	0–2
ESOPEC (experimental arm)	Adenocarcinoma	Peri-operative FLOT + esophagectomy	5.5 years	57%	51%	T1N1M0, T2-3N0-1M0	0–2
ESOPEC (CROSS ARM)	Adenocarcinoma	CRT + esophagectomy	3.1 years	51%	39%	T1N1-3M0, T2-4NanyM0	0–1
Neo-AEGIS	Adenocarcinoma	Peri-operative CHT + esophagectomy	4.0 years	55%	NA	T2-3N0-3M0	0–2
Matoska et al. (no induction arm)	Adenocarcinoma	CRT +/− esophagectomy	2.3 years	43%	31%	T1N1-3M0, T2-4NanyM0, TanyNanyM1 (oligometastatic)	0–3

NA = not applicable. No data was available for this datapoint.

## Data Availability

The data presented in this study are available on request from the corresponding author due to privacy restrictions. The data is not publicly available due to patient identifiers.

## Abbreviations

The following abbreviations are used in this manuscript:

EAC	esophageal adenocarcinoma
CRT	chemoradiation
CHT	chemotherapy
OS	overall survival
PFS	progression free survival
pCR	pathologic complete response
SCC	squamous cell carcinoma
FLOT	5-FU, leucovorin, oxaliplatin, docetaxel
FOLFOX	leucovorin, fluorouracil [5-FU], and oxaliplatin
DOC	docetaxel + oxaliplatin + capecitabine

## References

[1] American Cancer Society (2025). American Cancer Society Statistics Esophageal Cancer. https://www.cancer.org/cancer/types/esophagus-cancer/key-statistics.html.

[2] (2025). NIH Surveillance, Epidemiology, and End Results Program. Cancer Stat Facts: Esophageal Cancer. https://seer.cancer.gov/statfacts/html/esoph.html.

[3] van Hagen P., Hulshof M.C., van Lanschot J.J., Steyerberg E.W., van Berge Henegouwen M.I., Wijnhoven B.P., Richel D.J., Nieuwenhuijzen G.A., Hospers G.A., Bonenkamp J.J. (2012). Preoperative chemoradiotherapy for esophageal or junctional cancer. N. Engl. J. Med..

[4] Sheikh M., Roshandel G., McCormack V., Malekzadeh R. (2023). Current Status and Future Prospects for Esophageal Cancer. Cancers.

[5] Hoeppner J., Brunner T., Schmoor C., Bronsert P., Kulemann B., Claus R., Utzolino S., Izbicki J.R., Gockel I., Gerdes B. (2025). Perioperative Chemotherapy or Preoperative Chemoradiotherapy in Esophageal Cancer. N. Engl. J. Med..

[6] The National Comprehensive Cancer Network NCCN Clinical Practice Guidelines in Oncology: Esophageal and Esophagogastric Junction Cancers. Version 2.2025. https://www.nccn.org/guidelines/guidelines-detail?category=1&id=1433.

[7] Worrell S.G., Goodman K.A., Altorki N.K., Ashman J.B., Crabtree T.D., Dorth J., Firestone S., Harpole D.H., Hofstetter W.L., Hong T.S. (2024). The Society of Thoracic Surgeons/American Society for Radiation Oncology Updated Clinical Practice Guidelines on Multimodality Therapy for Locally Advanced Cancer of the Esophagus or Gastroesophageal Junction. Ann. Thorac. Surg..

[8] Stahl M., Walz M.K., Stuschke M., Lehmann N., Meyer H.J., Riera-Knorrenschild J., Langer P., Engenhart-Cabillic R., Bitzer M., Königsrainer A. (2009). Phase III comparison of preoperative chemotherapy compared with chemoradiotherapy in patients with locally advanced adenocarcinoma of the esophagogastric junction. J. Clin. Oncol..

[9] Carr R.A., Hsu M., Harrington C.A., Tan K.S., Bains M.S., Bott M.J., Ilson D.H., Isbell J.M., Janjigian Y.Y., Maron S.B. (2023). Induction FOLFOX and PET-Directed Chemoradiation for Locally Advanced Esophageal Adenocarcinoma. Ann. Surg..

[10] Yoon H.H., Ou F.S., Soori G.S., Shi Q., Wigle D.A., Sticca R.P., Miller R.C., Leenstra J.L., Peller P.J., Ginos B. (2021). Induction versus no induction chemotherapy before neoadjuvant chemoradiotherapy and surgery in oesophageal adenocarcinoma: A multicentre randomised phase II trial (NCCTG N0849 [Alliance]). Eur. J. Cancer.

[11] von Döbeln G.A., Klevebro F., Jacobsen A.B., Johannessen H.O., Nielsen N.H., Johnsen G., Hatlevoll I., Glenjen N.I., Friesland S., Lundell L. (2019). Neoadjuvant chemotherapy versus neoadjuvant chemoradiotherapy for cancer of the esophagus or gastroesophageal junction: Long-term results of a randomized clinical trial. Dis. Esophagus.

[12] Ho F., Torphy R.J., Friedman C., Leong S., Kim S., Wani S., Schefter T., Scott C.D., Mitchell J.D., Weyant M.J. (2021). Induction Chemotherapy Plus Neoadjuvant Chemoradiation for Esophageal and Gastroesophageal Junction Adenocarcinoma. Ann. Surg. Oncol..

[13] Peters G.W., Talcott W., Peters N.V., Dhanasopan A., Lacy J., Cecchini M., Kortmansky J., Stein S., Lattanzi S., Park H.S. (2023). Pre-operative chemoradiotherapy with or without induction chemotherapy for operable locally-advanced esophageal cancer. J. Gastrointest. Oncol..

[14] Rice T.W., Patil D.T., Blackstone E.H. (2017). 8th edition AJCC/UICC staging of cancers of the esophagus and esophagogastric junction: Application to clinical practice. Ann. Cardiothorac. Surg..

[15] Kaplan E.L., Meier P. (1958). Nonparametric Estimation from Incomplete Observations. J. Am. Stat. Assoc..

[16] Cox D.R. (1972). Regression Models and Life-Tables. J. R. Stat. Soc. Ser. B (Methodol.).

[17] Gray R.J. (1988). A class of K-sample tests for comparing the cumulative incidence of a competing risk. Ann. Stat..

[18] R Core Team (2025). R: A Language and Environment for Statistical Computing.

[19] Goodman K.A., Ou F.S., Hall N.C., Bekaii-Saab T., Fruth B., Twohy E., Meyers M.O., Boffa D.J., Mitchell K., Frankel W.L. (2021). Randomized Phase II Study of PET Response-Adapted Combined Modality Therapy for Esophageal Cancer: Mature Results of the CALGB 80803 (Alliance) Trial. J. Clin. Oncol..

[20] Reynolds J.V., Preston S.R., O’Neill B., Lowery M.A., Baeksgaard L., Crosby T., Cunningham M., Cuffe S., Griffiths G.O., Parker I. (2023). Trimodality therapy versus perioperative chemotherapy in the management of locally advanced adenocarcinoma of the oesophagus and oesophagogastric junction (Neo-AEGIS): An open-label, randomised, phase 3 trial. Lancet Gastroenterol. Hepatol..

[21] Matoska T., Banerjee A., Shreenivas A., Jurkowski L., Shukla M.E., Gore E.M., Linsky P., Gasparri M., George B., Johnstone C. (2023). Definitive Chemoradiation Associated with Improved Survival Outcomes in Patients with Synchronous Oligometastatic Esophageal Cancer. Cancers.

[22] Shimodaira Y., Slack R.S., Harada K., Chen H.C., Sagebiel T., Bhutani M.S., Lee J.H., Weston B., Elimova E., Lin Q. (2018). Influence of induction chemotherapy in trimodality therapy-eligible oesophageal cancer patients: Secondary analysis of a randomised trial. Br. J. Cancer.

[23] Ajani J.A., Xiao L., Roth J.A., Hofstetter W.L., Walsh G., Komaki R., Liao Z., Rice D.C., Vaporciyan A.A., Maru D.M. (2013). A phase II randomized trial of induction chemotherapy versus no induction chemotherapy followed by preoperative chemoradiation in patients with esophageal cancer. Ann. Oncol..

[24] Al-Batran S.E., Goetze T.O., Mueller D.W., Vogel A., Winkler M., Lorenzen S., Novotny A., Pauligk C., Homann N., Jungbluth T. (2017). The RENAISSANCE (AIO-FLOT5) trial: Effect of chemotherapy alone vs. chemotherapy followed by surgical resection on survival and quality of life in patients with limited-metastatic adenocarcinoma of the stomach or esophagogastric junction—A phase III trial of the German AIO/CAO-V/CAOGI. BMC Cancer.

[25] Janjigian Y.Y., Al-Batran S.E., Wainberg Z.A., Muro K., Molena D., Van Cutsem E., Hyung W.J., Wyrwicz L., Oh D.Y., Omori T. (2025). Perioperative Durvalumab in Gastric and Gastroesophageal Junction Cancer. N. Engl. J. Med..

[26] Shitara K., Özgüroğlu M., Bang Y.J., Di Bartolomeo M., Mandalà M., Ryu M.H., Fornaro L., Olesiński T., Caglevic C., Chung H.C. (2018). Pembrolizumab versus paclitaxel for previously treated, advanced gastric or gastro-oesophageal junction cancer (KEYNOTE-061): A randomised, open-label, controlled, phase 3 trial. Lancet.

[27] Yee E.J., Read J., Ziogas I.A., Stuart C.M., Olsen J., Kim S.S., Mitchell J.D., Meguid R.A., McCarter M.D., Mungo B. (2025). More May Not Be Better: Comparison of Oncologic Outcomes Following Induction Chemotherapy Plus Chemoradiation and Chemoradiation Alone for Esophageal Adenocarcinoma. J. Surg. Oncol..

[28] Leong T., Smithers B.M., Michael M., Haustermans K., Wong R., Gebski V., O’Connell R.L., Zalcberg J., Boussioutas A., Findlay M. (2024). Preoperative Chemoradiotherapy for Resectable Gastric Cancer. N. Engl. J. Med..

[29] Lin E.W., Karakasheva T.A., Lee D.J., Lee J.S., Long Q., Bass A.J., Wong K.K., Rustgi A.K. (2017). Comparative transcriptomes of adenocarcinomas and squamous cell carcinomas reveal molecular similarities that span classical anatomic boundaries. PLoS Genet..

[30] Liu S., Chen B., Zhu Y., Wang S., Cheng X., Wang R., Hu Y., Liu H., Li Q., Zhang L. (2025). Induction chemotherapy plus chemoradiotherapy in esophageal cancer: Long-term results and exploratory analyses of a randomized controlled trial. Oncologist.

[31] Olson J. (2024). Phase II Study of Induction FLOT Followed by Neoadjuvant Chemoradiation in Patients with Resectable Adenocarcinoma of the Esophagus or Gastroesophageal Junction. https://clinicaltrials.gov/study/NCT04028167.

[32] Slagter A.E., Jansen E.P.M., van Laarhoven H.W.M., van Sandick J.W., van Grieken N.C.T., Sikorska K., Cats A., Muller-Timmermans P., Hulshof M., Boot H. (2018). CRITICS-II: A multicentre randomised phase II trial of neo-adjuvant chemotherapy followed by surgery versus neo-adjuvant chemotherapy and subsequent chemoradiotherapy followed by surgery versus neo-adjuvant chemoradiotherapy followed by surgery in resectable gastric cancer. BMC Cancer.

[33] Lin S.H., Hobbs B.P., Verma V., Tidwell R.S., Smith G.L., Lei X., Corsini E.M., Mok I., Wei X., Yao L. (2020). Randomized Phase IIB Trial of Proton Beam Therapy Versus Intensity-Modulated Radiation Therapy for Locally Advanced Esophageal Cancer. J. Clin. Oncol..

[34] Puckett L. (2025). Pulso Trial: Pulsed Low-Dose-Rate (PLDR) Radiation Chemoradiation (CRT) vs. Standard CRT for Esophageal Cancer. https://clinicaltrials.gov/study/NCT06906887.

